# Comparing the Effects of Two Feeding Methods on Metabolic Bone Disease in Newborns With Very Low Birth Weights

**DOI:** 10.5539/gjhs.v8n1p249

**Published:** 2015-05-28

**Authors:** Asghar Lotfi, Kobra Shiasi, Razieh Amini, Mohammad Jahangiri, Mohammad Reza Sharif, Hossein Akbari, Hamidreza Talari, Zahra Hajmobini, Kamran Hami, Hamed Haddad Kashani

**Affiliations:** 1 Department of Pediatrics, Kashan University of Medical Science, Kashan, Iran; 2 Research Center of Biochemistry and Nutrition in Metabolic Disorder, Kashan University of Medical science. Kashan, Iran; 3 Department of Nursing, Najafabad Branch, Islamic Azad University, Najafabad, Iran; 5 Department of Biostatics and Epidemiology, School of Public Health, Kashan University of Medical Science, Kashan, Iran; 6 Department of Radiology, Kashan University of Medical Science, Kashan, Iran; 7 Anatomical Sciences Research Center, Kashan University of Medical Sciences, Kashan, Iran

**Keywords:** Newborns with very low birth weights (VLBW), Metabolic bone disease (MBD), feeding methods, Preterm formula

## Abstract

**Introduction:**

Bone metabolic disease is an important issue in newborns with very low birth weight. The 80 percent of the transport of calcium (Ca) and phosphor (P) from mother to fetus takes place in the third trimester of pregnancy. This transport process is impaired with the preterm delivery of the newborn. On the other side, breast milk and formula are not competent resources to supply sufficient amounts of Ca and P to meet the requirements of the preterm newborn, thereby a greater reduction in the storage of these minerals. The current study has been done with the purpose of comparing the effects of two feeding methods on the indices of metabolic bone disease in newborns with very low birth weights (VLBW).

**Materials and Methods:**

The study design was cohort and the study was done on a total of 58 newborns with very low birth weights in Kashan Shahid Beheshti Hospital. The newborns were divided into two groups with 29 placed in the group of alternate feeding on preterm formula (preNan) and the other 29 placed in the group of breast milk and preterm formula (preNan). Eventually, the indices of bone metabolic disease were measured in both groups and were statistically analyzed.

**Results:**

The difference between the mean serum levels of Ca in different weeks and also between the two groups were significant (p=0.001). However, the changes in mean serum level of P in the two groups were not significant (P=0.151). The comparison of serum levels of alkaline phosphatase between the two groups indicated that their difference was significant and that they had been influenced by the feeding method (P=0.001). The serum level of bicarbonate, when compared between the two groups, was found to make a significant difference (P=0.001). The difference between the two feeding methods in precipitating rickets and osteopenia was not significant.

**Conclusion:**

According to the findings of current study, feeding on preterm formula (preNan) is associated with better and more desirable results, though the occurrence of rickets in the two groups had no significant difference.

## 1. Introduction

The birth rate of neonates with very low birth weight (i.e. birth weight lower than 1500 g) is about 1.47% of total live births (Kliegman, 2011). Although these neonates account for only 1 to 2% of all deliveries, they comprise a large proportion of infant and neonatal mortality and its short-term and long-term consequences as well (Kliegman, 2011). Calcium and phosphorus are the most important constitutive elements of human skeleton, as 99% of calcium and 80% of phosphorus in human body are present in skeleton in form of microcrystalline hydroxylapatite (Ca_5_(PO_4_)_3_(OH)) (Vachharajani et al., 2009). It is recognized that 80% of Ca and P transport from mother to fetus occurs over the third trimester of pregnancy and with preterm deliveries such transportation is impaired. As a result, preterm infants lose a rich source of Ca and P which is sufficient to mineralize their developing skeleton, and hence reduction in bone mineralization ensues (Kliegman, 2011; Namgung & Tsang, 2003). The total body calcium reaches from 5 grams at the end of the second trimester to 30-35 grams at term. Meanwhile, the maximum rate of increase hits 120-160 mg/kg/d for Ca, 60-75 mg/kg/d for P and 2.5-3.4 mg/kg/d for magnesium. Such Mg comprises 60% of body magnesium at birth (Vachharajani et al., 2009; Fewtrell, 2011). On the other hand, breast milk and infant formula are not sufficient sources to meet the nutritional requirements of preterm infants, which in term aggravate their deficiency of such elements. The leading nutritional etiology of metabolic bone disease (MBD) in preterm infants is phosphorus deficiency (Vachharajani et al., 2009). Preterm infants who are solely nourished with breast milk will develop hypophosphatemic hypercalcemia (Vachharajani et al., 2009). The low amount of phosphorus in unfortified infant formula leads also to hypophosphatemia, hypercalcemia and hypercalciuria (Vachharajani et al., 2009). Although preterm infants need vitamin D additive supplements, the metabolic bone disease can occur even with normal or high serum levels of 1,25-dihydroxy vitamin D3 (Laing et al., 1985; Koo et al., 1982). The incidence of MBD also depends on the way of feeding preterm infant and varies from 40% in exclusive breastfeeding to 20% in alternative breastfeeding and formula-feeding with preterm infant formula and 15% in exclusive preterm infant formula-feeding (Vachharajani et al., 2009). The suggested ways of feeding preterm infants and even neonates with birth weights lower than 2 kilograms include exclusive formula-feeding with special preterm infant formulas or breastfeeding with the breast milk fortified with supplements which fulfill the special and extra requirements of these infants of calorie, calcium and phosphorus (fortifier) (Takada et al., 1992; Trindade, 2005; Lapillone et al., 2004; Harrison et al., 2008). To resolve this conflict, it is recommended in some resources to feed the baby with breast milk and preterm infant formula every other meal, which is conducted in some centers. In this study, we have decided to compare the two aforementioned ways of feeding the infant and their effects on the prevention of metabolic bone disease in preterm infants.

## 2. Methods and Materials

This study is a cohort study of newborns with weights less than or equal to 1500 grams who were admitted to neonatal intensive care unit(NICU) of Kashan Shahid Beheshti Hospital and qualified for the inclusion criteria of current study. According to previous studies and considering the confidence interval, the sample size was estimated to be 52. The data was collected using the convenience nonpropabilty sampling method and qualified newborns were placed in groups 1 or 2 on the basis of their date of birth and nourishment with either breast milk or formula, until each group attained sufficient size. A questionnaire was designed to record each newborn’s data including personal identity, file number and address, and then birth weight, gestational age, multiple birth, the cause of preterm labor, delivery type, previous history of premature birth or low birth weight (LBW), presence of hyaline membrane disease (HMD), treatment with surfactant, history of total parenteral nutrition (TPN) and its period, presence of intraventricular hemorrhage (IVH) and its clinical course, and day of beginning oral feeding. The study lasted 12 weeks and all the newborns were followed through this period. These newborns were also blood sampled at least 3-6 times in addition to the first time. During the period of hospitalization, serum levels of Ca, P, alkaline phosphatase (ALP) and arterial blood gas (ABG) were measured and recorded weekly, but after discharge, these measurements were repeated every 2-4 weeks. Between 8-12 weeks of age, a chest x-ray and left wrist x-ray were performed for each newborn to the purpose of radiologic diagnosis of metabolic bone disease. (In case of an ALP level greater than 1000, the imaging is performed for the timely diagnosis of the disease.) At last, the images are interpreted by two experienced radiologists separately without being informed of laboratory results and the other radiologists’ interpretation. The radiological reports were classified into three classes “for convenience” as following; Early Rickets is the initial presentation of rickets with the absence of any provisional zone of calcification (PZC) or the presence of only one of the radiologic manifestations of rickets including cupping, frying, and widening. Rickets is established and advanced rickets with full presentation reported by both of the two radiologists, and Osteopenia is reduced density of bone mineral tissue noted in radiologic reports. Following data collection, the frequencies of bone metabolic diseases, i.e. rickets, osteopenia, and 5bone fracture, over the different weeks of therapy were calculated and tested by Chi-squared and Fischer’s exact tests. The analysis of biochemical parameters including Ca, P, ALP, and HCO_3_ was also done using the T test and Mann Withney U test. Furthermore, the generalized estimating equations (GEE) were employed to determine the effect of time on changes in biochemical parameters of the disease.

## 3. Results

In this study, 58 newborns with gestational ages of 30.58±2.65 and a mean weight of 1265±262 gr were studied, 29 thereof were placed in the group of preterm formula feeding and the other 29 in the group of alternate feeding with breast milk and preterm formula. The mean age of initiating efficient oral feeding ranged from 1 to 90 days and the mean age of initiating oral feeding was 12.3±14.48 days. The mean age of initiating oral feeding in the first and second group were assessed to be 17.26± 18.67 and 8.26±9.05 respectively. In the second group, the mean period of continuing breastfeeding was 83.67±74.01 days with the minimum and maximum being 20 days and to the end of the study, respectively. Among the studied newborns, 32.7% were found to be small for their gestational age (SGA) and 67.3% appropriate for their gestational age (AGA). The two groups were matched and there was no significant difference between them in terms of multiple birth, delivery type and so on. The incidence of intraventricular hemorrhage (IVH)-assessed by ultrasound of the brain- was 37.9% (11 newborns) in the first group with 62.1% (18 newborns) lacking IVH. In the second group, IVH was found to be present in only 2 newborns with the remaining 27 lacking IVH. There existed a significant difference between the two groups. (P value= 0.05). The mean weight of the first and second group was 1194±251.5 g and 1340±171.6 g respectively with their difference being significant. (P value= 0.013). The mean height of the first and second group was 38.06±2.49 cm and 39.48±2.69 cm respectively, the difference of which was significant as well. (P value= 0.043). The mean head circumference of the first and second group was 28.02±2.06 cm and 228.95 ±1.68 cm respectively, but their difference was not significant (P value= 0.065). The average serum calcium, assessed over the different weeks of life, was compared between the two groups by statistical analysis and GEE test and there was found consequently a significant difference between them with regard to their different methods of feeding. (P value= 0.001) The difference was also chronologically significant over the different weeks (P value < 0.001). The average serum phosphorus between the two groups was not significant with regard to their feeding methods, but it was chronologically significant in the course of study (P value= 0.001). The serum phosphorus level diminished in both of the groups over time reaching its minimal level in the fourth week of life and thenceforth increased gradually. There was no significant difference between the two types of milk, i.e. breast milk and formula, according to the graph, but the time had been influential. The decrease in Phosphorus was not significant (P value= 0.15). It gradually decreased to its minimal level around the first month of age and increased with a roughly equal gradient. The comparison of ALP levels over the different weeks indicated a significant difference between the two groups in the course of study with the feeding method being influential (P value 0.001). The changes were also chronologically significant (P value < 0.001).

**Table 1:** The frequency of osteopenia compared between the two groups

Groups	Preterm formula	Breast milk + Preterm formula	P value
Osteopenia			
Yes	17 (58.6%)	22(75.9%)	0.162
No	12 (41.4%)	7 (24.1%)	

As it is clear from Table 2, in the first group -preterm formula-, 17 newborns were without and 12 newborns with osteopenia whereas in the second group-breast milk and preterm formula-, osteopenia was not found in 22 of the newborns but it was present in 7 of them.

**Table 2:** The prevalence of rickets in the two groups

Groups	Preterm formula	Breast milk + Preterm formula	P value
X-ray interpretation			
Normal	19 (65.5%)	17 (58.5%)	0.927
Earl Rickets	6 (20.7%)	8 (27.6%)	
Rickets	4 (13.8%)	4 (13.8%)	

The prevalence of rickets, as shown by the X-rays of the newborns of both groups, was compared between the two groups (Table 2). In the preterm formula group, 19 newborns (65.5%) were normal, 6 (20.7%) diagnosed with early rickets, and 4 (13.8%) diagnosed with established rickets, while in the second group-breast milk and preterm Formula-17 newborns (58.5%) were normal, 8 (27.6%) diagnosed with early rickets, and 4 (13.8%) diagnosed with established rickets. It is to be noted that the different feeding methods made no significant distinction between the prevalence of rickets in the two groups.

**Table 3:** The radiologic findings in studied infants

X-ray finding		Number	Percent (%)
Frying		4	6.9
Widening		9	15.5
Lack of PZC	Complete	15	25.8
	Incomplete	3	5.2
Cupping		9	15.5
Isolated osteopenia		9	15.5

Overall, among all the studied newborns, 22 were diagnosed with rickets, the 8 of which had full presentation of rickets including frying, cupping and widening, but the remainder demonstrated only the initial presentations of rickets –i.e. early rickets- like lack of PZC or isolated frying.

As it is demonstrated in Table 4, there was found no relationship between the feeding method and the incidence of rickets in the two groups and the difference between them was not significant. (P value= 0.927).

**Table 4:** The relationship between feeding method and incidence of rickets in studied newborns

Final Diagnosis	Normal	Early Rickets	Rickets	P value
Feeding method				
Preterm formula	65.5%	20.7%	13.8%	0.927
Breast milk + Preterm formula	58.6%	27.6%	13.8%	

Dividing the studied newborns into two weight groups, i.e. one with weights less than or equal to 1200 g and the other with weights greater than 1200 g, the incidence of rickets was examined in both the groups and it was revealed that the weight influences the incidence of rickets, thereby the greater incidence of bone disorders including early rickets and rickets in newborns weighed less than or equal to 1200 g than that of the other group with their difference being significant. (P value= 0.036). The total number of studied newborns was 58 and 22 of them weighed less than or equal to 1200 g, 12 (54.6%) of which were diagnosed with some degree of rickets with 6 (27.3%) of them presenting with early rickets and the other 6 with full rickets. Among the other 36 newborns of the study, weighing greater than 1200 g, solely 2 (5.6%) of them had full rickets and 8 (22.2%) had early rickets with the total being 10 newborns (27.8%) showing some degree of rickets. (P value= 0.036).

## 4. Discussion

The mean weight and gestational age of the studied newborns were 1265±22 g and 30.58±2.65 weeks respectively. The mean weight can be influenced by factors like genetic, race, nutrition, and various environmental factors. In a study done by Koo and colleagues, 19 infants were followed from the time of birth to 10 weeks of age with their weights ranging between 740 and 1296 g with an average of 1100 g. It was demonstrated that the newborn’s birth weight (BW) and gestational age (GA) are more appropriate factors in foretelling the occurrence of metabolic bone disease (MBD) than biochemical factors, so that 6 newborns of the 8 who weighed less than 1000 g had radiological changes and the newborns who showed signs of skeletal demineralization, had lesser gestational ages and birth weights (Koo et al., 1982, 1989). In our study, the birth weight and gestational age similarly play an important part in predicting the occurrence of BMD, as there was a significant relationship between the GA of 28 weeks and the occurrence of rickets. Early rickets occurred only from 28 to 32 weeks of age and not after 32 weeks and osteopenia was related to a GA less than 28.5 weeks as well. Furthermore in our study, there was obtained a cut-off point of 1200 g for birth weight, so that the newborns weighing less than 1200 g developed rickets and early rickets more frequently than those weighing greater than 1200 g and the difference between the two groups were significant. (P value= 0.036) (Table 5). In another study conducted by Chuhan, the risk of developing MBD was inversely related to GA and BW, while directly related to postnatal complications (Chauhan et al., 2011). In our study, the frequency of rickets among the preterm infants was 37.9% (32 of a total of 58) and the frequency of osteopenia was 32.7% (19 of a total of 58), which is in analogy with other studies. It is known that the postnatal complications affect the occurrence of BMD, of which hyaline membrane disease (HMD), treatment with surfactant, total parenteral nutrition (TPN) and intraventricular hemorrhage (IVH) were studied in current study and the sole complication which could significantly affect the development of BMD was HMD. According to Nelson’s textbook of pediatrics, a total parenteral nutrition (TPN) of more than 4 weeks and administration of drugs like diuretics and corticosteroids are among the risk factors for MBD (Aiken et al., 1993). The reasons why TPN did not produce a significant effect in our study could be errors in the process of data collection or TPN periods of less than 4 weeks in the majority of infants of our study. The first group (preNan) had a mean serum Ca level of 8.88±1.08 and the second group (preNan + breast milk) had a mean serum Ca level of 8.87±0.86, the both of which are within the normal range as expected, because the serum Ca in BMD usually lies within or lower than the normal range.

**Table 5:** The relationship between the weight of newborns and incidence of rickets in the two weight groups

Final Diagnosis	Normal	Early Rickets	Rickets	P value
weight				
≤1200 g	45.5%	27.3%	27.3%	0.036
>1200 g	72.2%	22.2%	5.5%	

One of the other important parameters in foretelling the occurrence of BMD is serum P level. In our study, all the infants with rickets had a serum P level of lower than 5.6. The mean serum P level among the both groups of the study showed no significant difference. This finding is analogous with the findings of other studies and statements of the reference books. In Nelson’s textbook of pediatrics, a serum P level of less than 5.6 is regarded as a risk factor for developing BMD, which is confirmed also by our study (Kliegman et al., 2001). In our study, high levels of ALP was not found very frequently and moreover, serum ALP levels in infants with rickets or osteopenia had no significant relationship with those of the normal infants, which is in contrast to the findings of other studies. In the study of Mitchell indicated that the serum ALP level has an inverse significant relationship to BW, GA, and serum P level and that a serum ALP level of more than 800 is an indication for screening the infant for MBD and probably initiating the nutritional interventions (Mitchell et al., 2009). Eventually, Backstorm stated that a serum ALP level of more than 300 in three months of age-adjusted with gestational age can predict the rate of radiological osteopenia with a sensitivity of 88% and a specificity of 71%. One of the factors affecting the occurrence of MBD is efficient oral feeding, so that the sooner does the oral feeding begin, the lesser will be the risk of bone complications (Backströma et al., 1999). In our study, the mean age of initiating efficient oral feeding was 17.26±18.67 days in the first group and 8.26±9.05 days in the second group and overall, among the studied infants ranged from 1 to 90 days. The other studies also confirm the finding of scientist that infants with radiological changes had begun oral feeding later than infants without radiological changes (Koo et al., 1993; Aiken et al., 1993; Kleinman et al., 1998; ESPGAN Committee, 1987).

## 5. Conclusion

In this study, there was found no difference between the two nutritional groups in terms of the occurrence of bone disorders (Rickets). The frequency and severity of metabolic bone disease (MBD) was in inverse relationship to gestational age and birth weight. The sooner initiation of oral feeding reduces the risk of MBD. Infants with HMD are more prone to MBD.
